# Financial Budgets of Technology-Based SMEs From the Perspective of Sustainability and Big Data

**DOI:** 10.3389/fpubh.2022.861074

**Published:** 2022-05-04

**Authors:** Guobiao Zhang, Tao Wang

**Affiliations:** ^1^School of Business, Zhejiang University City College, Hangzhou, China; ^2^School of Business, Zhejiang University City College, Hangzhou, China

**Keywords:** small and medium sized enterprises, big data, sustainability, finance, financial budgets, technology adaptation

## Abstract

In this contemporary world, the words data and sustainability play a crucial role in determining the financial budgets of small and medium-sized enterprises (SMEs). Usually, it is stated that the survival of small and medium-sized enterprises (SMEs) is directly proportional to the growth and sustainability factor of the nation. The economic sustainability of a nation is dependent on appropriate functioning of SMEs. Any kind of direct impact on the working of SMEs will have its impact on the whole economy of a nation. There are different factors such as lack of financial capacity, low market demands, restrictions with regard to the capital, and barriers in the supply chain that affect the sustainability of SMEs worldwide. Nevertheless, small, and medium sized enterprises around the world are greatly investing on skills, innovation, and other capital related resources to mark up the demands of the external market. The main objective of this study is to examine the financial budgets of technology-based SMEs from the perspective of sustainability and big data. For this, the study collects data through a questionnaire from 1,800 Small and Medium Sized Enterprises. Based on a detailed and careful examination of the data, only 1,400 of the responses received were considered valid (79.75%). To test the hypothesis stated, the study employs structural equation modeling. This will help the researcher to examine the direct effect of financial budget and technology adaption of SMEs from the perspective of sustainability and big data. Results of the study stated that SMEs sustainability and big data are directly and positively related to the financial budget planning of technology-based SMEs. The study also found that big data plays an important role in the businesses, specifically for their own growth.

## Introduction

Recently, businesses across the world are facing extreme sustainability issues and thereby adversely impacting the economy of the nations. Due to the current societal circumstances, most of the businesses are enforced to formulate new ideas to deal with the problems associated with the pressurized societal situation. Especially, Small and Medium sized Enterprises (SMEs) recorded the most pressing difficulties including financial and Non-financial. There are other factors like limited market demand, supply chain disruption, and trade discontinuation that will impact the economy of the nation (1). The current environmental situations in the world critically affected the economy of the nation, nevertheless, the most affected sector was identified as Small and Medium sized Enterprises (SMEs) (2).

Very recently, (3) provided evidence stating that SMEs act as the major driving force in increasing the growth of the nation. SMEs also create employment opportunities, open markets for economic sustainability, and develop trade liberalization. Because of financial and economic crisis, several small and medium sized enterprises are facing challenges. Most of the SMEs are failing to contribute the economic development of their nation. Several studies, for instance (4, 5), have revealed that SMEs are the gravest hit industries, especially during any type of crisis. It was noted that these SMEs knowledge level was low, susceptible, less reliant, and so on. Moreover, SMEs were greatly dependent on financial agents who support them during a crisis (6). Several studies also recorded that SMEs are facing multiple challenges with regard to financial obligation such as paying credit to the financial institutions, identifying shortages in the inventory, and identifying shortage in the operations.

To achieve the goal of sustainability, it becomes equally important for the SMEs to predominantly survive and contribute to the total economy of the country. Although there exist several ways that hinder the growth of SMEs, it becomes crucial for an SME to survive by itself and contribute to the whole economy of the country (7). Despite SMEs have sorted their own methods/ways to deal with survival, it had affected their businesses in a positive and negative manner. Usually, owners of the businesses are concerned about the operations of the SMEs, which included financial budget activities, financial disruption activities, creating unemployment, creating social imbalance, creating security imbalance, and planning the whole production. It was also observed that these operations had had a deep impact on the society (8).

Preliminary studies of SMEs sustainability primarily focused on exploring factors that affected the growth and development of small and medium sized enterprises. Factors such as adopting technology in the SMEs, implementing financial budgets, appropriating financial planning, effective big data management, and so forth affected the growth and functioning of SMEs (9). A growing number of literatures has proved that technology-based SMEs perform well in terms of dealing with sustainability and big data. It was also found that there is a positive relationship between adapting technology and sustainability of SMEs. From the above statement, it can be deduced that the technology-based functioning SMEs find advantages in the market and thus moving toward the path of sustainability (10). Although there exist several studies exploring the relationship between finance and the growth of SMEs, so far, no study has tried to analyze the financial budgets of technology-based SMEs from the perspective of sustainability and big data.

Past studies confirmed that data is regarded as one of valued assets for any business, and it's been increasing rapidly day-by-day. This valued asset is popularly termed as big data that comes with 3 peculiar features. These three features are identified as having high volumes of data, variety of data types, and velocity to process the data. Almost all the organizations in the world are striving hard to discover all the possible ways to utilize this big data and develop their business. Majority of the studies confirmed that big data is quite important in the growth and development of a business (11). It was also added that, both large and small organizations are focusing on accurate and valuable data to make some important decisions related to business development. Likewise, few other studies demonstrated that big data supports SMEs to reach out to their target audience, understand their preferences, identify their needs, and then meet them in new ways (9, 12). Hence, it can be understood that big data plays a significant role in the growth of an SME, which is why SMEs should adopt big data in their business. Therefore, this study focuses on examining the financial budgets of technology-based SMEs from the perspective of sustainability and big data, which are considered as the backbone of a nations' economy. The other objectives of the study are to improve the business process of SMEs by adopting new technologies and planning for financial budgets, which will effectively promote the growth of SMEs. This study will address the existing research gap by examining the process of planning financial budgets to the technology-based SMEs through the perspective of big data and sustainability. It will also examine the relationship between finance and SMEs; technology and SMEs; big data and SMEs; and try to understand this complex relationship from the perspective of SME sustainability.

## Literature Review

### Relationship Between Finance and SMEs

The growth of small and medium sized enterprises is directly related to the aspect of finance. Innovations with regard to finance, especially in the smooth functioning of SMEs play a critical role in various forms. In order to perform well, external and internal financial resources are necessary within an SME (13). Generally speaking, the performance of a firm is predominantly determined by intellectual resources and other major decisions. Moreover, any firm seems to face external and internal constraints (14). Past studies advocated that SMEs receive financial support from different traditional sources like merchant banks, development banks, commercial banks and so on. Most of the existing financial institutions were established to maintain the financial budgets of private organizations. For a smooth flow of business, these banks serve them with their effective channels (15). To manage the business in a much effective manner, every organization need loans from bank. From the above it can be understood that there is a direct relationship between credit of banks i.e.,' bank loans and the performance of a company, be it large or small and medium sized enterprises. The economic activity between a bank and an SME, is thus correlated.

Recent studies discussed about the effectiveness of the integration of e-commerce within Small and Medium sized Enterprises and found that latest technologies like mobile banking permits many SMEs the flexibility to settle down their financial transactions through the application (16). These new technologies also help the businesses to effectively perform financial transactions and reduce costs. It was also found that adopting mobile banking services within the small and medium sized enterprises will improve the diversity of business, reduce the cost, maintain the security, provide settlements with regard to finance. To perform monetary transactions, mobile commerce technology uses computer networks and other wireless networks. Previous studies observed that there has been a steady increase in the number of people using mobile applications and devices to buy products/services online. Using smartphone technology within the businesses can reduce the normal risks associated with finance. Hence, the study proposes the following hypothesis:

H1: There is a positive relationship between financing and SMEs sustainability.

### Relationship Between Technology and SMEs

Previous studies have shown that technology plays a crucial role in the effective functioning of an enterprise. Adapting to the innovative technology will permit the business entity to establish their brand in the market. It will also boost the competitor factor within the SMEs in the global market (17). As a result of globalization, the economic and financial condition of the SMEs are continuously persisting in terms of attaining large economy scale. Moreover, the revolution created by technology has led to several problems addressing the SMEs and the owners of SMEs had to respond to those issues. Interestingly, it was found that innovations in technology enhanced the business of SMEs and as a result SMEs witnessed a long-term growth. Hence, from this perspective it becomes important to understand the role of technology in achieving SMEs sustainability (18, 19).

Few other studies stressed that certain SMEs export their products/services at international level. They broaden their market with the help of innovative technologies. Technology based SMEs focused on pushing themselves to enter international market and expand their potential. When the SMEs are exposed to the overseas market, equivalently they et an opportunity to expand themselves and efficiently communicate, transport, and trade their services (20–22). Above all, advanced technologies enhanced the internal and external communication process from the organization perspective. It also boosted the competitiveness among SMEs by improving the quality of goods and services. With the advancement of technology, supply chain linkages has also improved by aiding new services.

Another important aspect of using technology in SMEs is that it gives excellent outcome in the market. Use of advanced technology strengthen the condition of firms in the markets at international and national level (23). Recent studies found the strong appeal on adapting technology within the SMEs. For various reasons like social culture, technology adaptation, economic structure, and political condition of nation, sometimes the business can be prolonged. Numerous studies discussed the advantages of implementing technologies in SMEs (24–26). Nevertheless, few other studies aimed to focus on adopting technology within small and medium sized enterprises. Both developing and developed countries are interested in adopting technology to the SMEs, which will enhance the performance of firms. Innovations in technology will enhance the firm performance, thereby making SMEs sustainable. Hence, it can be commented that technology-based SMEs are critical in developing the economy of nation. Thus, the study proposes the following hypothesis:

H2: There is a positive relationship between adopting technology and SMEs sustainability.

### Big Data and SMEs

There exists a considerable body of literature on the importance of data within the companies. Data is regarded as an important asset of SMEs. SMEs across the world are exploring different ways to tackle the data, which is usually called as big data. Big data is not only used in large multinational organizations, but also in SMEs. With the aim of improving the quality of a business, SMEs make use of big data to make accurate and quick decision in the need of hour (27, 28). The literature review shows that the use of big data in the business will enhance the whole process of business, and for this reason, SMEs are thinking on adopting big data to businesses (29–31). The use of big data technologies will help SMEs in deriving market value. The best way to deal with the challenges in the business is to adopt big data in SMEs (27). However, researchers have shifted their focus on big data, on discovering the benefits of big data.

A series of recent studies has indicated that multinational organizations are buying big data. Even startups are purchasing big data technologies to understand the new market, new clients, new business and so forth. Big data will also help SMEs to cut down the costs of business and enter the market. Transforming the big data to business applications will cost them real difficulty. Investing capital in the big data hardware and software is high. Few other studies revealed that technology adaptation, financial innovations, and big data are regarded as the strong pillars of SME (14, 16). In fact, organizations that use big data can increase their productivity and growth, which will improve the performance of enterprises. It will also help SMEs to understand the behavior of the market. Therefore, the study proposes the following hypothesis:

H3: There is a positive relationship between Big Data and SMEs sustainability.

H4: Big Data increases the growth and development of SMEs.

H5: Innovative Finance, Technology adaptation and Big Data technologies are essential in SMEs sustainability.

## Methodology

The current study mainly concentrates on examining the financial budgets of technology-based SMEs from the perspective of sustainability and big data. It also examines the relationship between innovative finance and SMEs, adapting new technologies within the SMEs, adopting to big data technologies from the perspective of SME sustainability. The analysis of the current study has been conducted through secondary literature and primary analysis as well. The study has developed hypothesis based on the analysis and review of the secondary literature. The formulated hypothesis is tested using a structured questionnaire. Thus, the current study gathers all the required data using questionnaire and hence the study is descriptive. Using the questionnaire's results the researcher will evaluate the hypothesis. This method involves describing the objectives, define the population, select the sample, and later interpret the data and results. Population is a complete set of individuals who are bound together by certain common characteristics. Population is of two types- target and accessible. Target population consists of those whom the researcher wishes to generalize the findings of the study. In the current study the total population include 7.2 million SMEs in China. From the total population, the study derives the required sample. The term sample includes the individuals selected to participate in the study. These selected participants are popularly known as respondents/subjects of research. Sampling methods are of two types–probability/random sampling and Non-probability. To carry out the research, this study employs random sampling method. At times, researchers also tend to use purposive sampling with the aim of obtaining required data from certain area of research.

For the purpose of fulfilling the objectives of the study, the study selects the sample consisting of 5,210 SMEs located in China. This study focused on those SMEs in China that were relatively easy to connect using an electronic channel. Prior to the administration of the questionnaire prepared, consent from the SMEs was imitated as the first step. Only after the participants acknowledgment, the questionnaire link was distributed to the selected sample. Out of 5,210 sample, around 4,000 of them acknowledged and agreed to participate in the study. So, in total 4,000 SMEs sent their prior consent, out of which only 2,000 were considered valid. Based on the analysis of the secondary literature, the researcher prepared a structured questionnaire, which addressed the factors like innovations in finance, adapting new technologies within the SMEs, adopting big data within SMEs from the perspective of SME sustainability. This structure questionnaire will allow the selected respondents to share their views, opinions, beliefs, attitudes, and values on the research topic. The data was collected during the period of November 2021 to December 2021 and sample of 3,000 was considered along with their responses. Table 1 presents the technical details of the research. In total, 1,800 responses were collected back and after the screening process, data of 1,400 respondents were finalized and considered for further analysis. Both primary and secondary data is collected to find out the results. The primary data is collected using the structured questionnaire designed to collect the required data from the participants. The collected data will help the researcher to examine the financial budgets of technology-based SMEs from the perspective of sustainability and big data. The researcher collects the secondary data required for the study from research articles and other journals. Finally, all gathered information was collected and analyzed to answer the research purpose.

**Table 1:** Research details.

**Headers**	**Comments**
**Sector**	Small and Medium sized Enterprises (SMEs)
**Location**	China
**Methodology**	Questionnaire–Structured in nature
**Sampling Technique**	Technique of Random Sampling
**Selected respondents**	1,800 (1,400)
**Period of data collection**	November 2021 to December 2021

**Figure 1:**
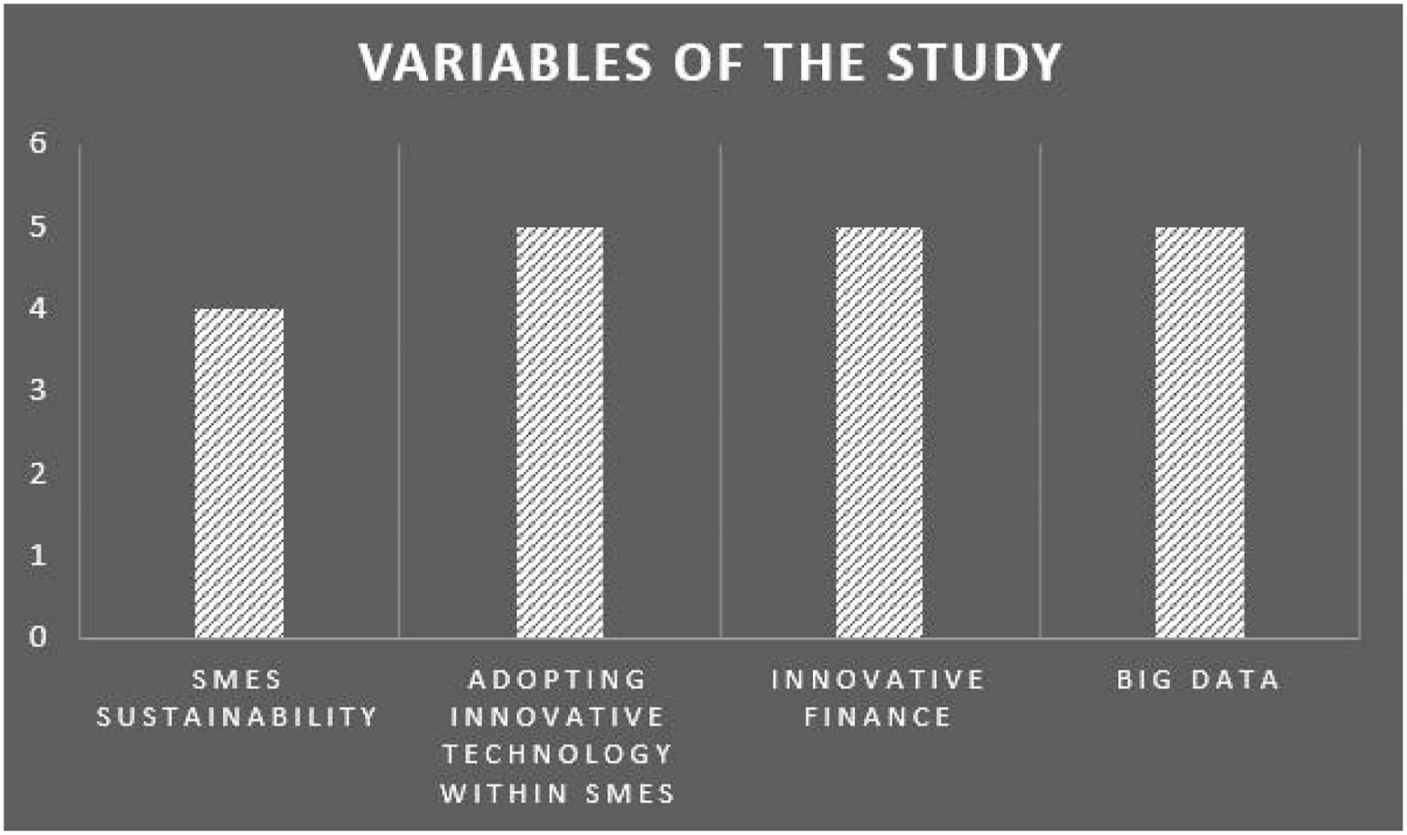
Graphical representation of the identified variables in the study.

The present identifies different variables like innovative finance, innovative technology, SMEs sustainability, and Big Data technology along with the measurement model. This model is presented in Table 2 and its graphical representation is presented in Figure 1.

**Table 2:** Variables of the study.

**Name of the Variable**	**Variable definition**	**Items (5-point likert scale)**
SMEs sustainability	Enhance services to customers, improve supply chain linkage, and strengthen their knowledge of market and trade relationships	4
Adopting innovative technology within SMEs	Adopting innovative technological advancements within the small and medium sized enterprises and large multinational organizations will help them to enhance their services to customers, improve supply chain linkage, and strengthen their knowledge of market and trade relationships.	5
Innovative finance	Innovative ways to manage financial products and services for effective operation of a business.	5
Big data	Big Data refers to massive volumes of data, which expands the business with new opportunities and challenges	5

To analyze the data collected, the researcher employs SPSS and AMOS −26.0 version. To study the relationship between the variables identified, the study adopts structural equation modeling. Furthermore, the selected SMEs will be able to provide accurate and precise information on the questions covered in the questionnaires related to the research topic. As the SEM method is highly resilient and efficient in modern times, the researcher decided to use the same. This method will help the researcher to examine the relationship between the variables identified by testing the proposed hypothesis. This method also focuses on the appropriateness of the results and hence was taken into consideration.

Although the owners of SMEs were reluctant to share the details of firms such as firm performance, innovations in finance budgeting, adopting innovative technologies, and adopting big data technologies, from the perspective of SME sustainability initially, later, they understood the need to evaluate the financial budgets of technology-based SMEs from the perspective of big data and sustainability and thereby opened, which helped the researcher to record the accurate data. Past researchers also tried to measure the sustainability of SMEs, which were in line with the opinions of the respondents, marked in 5-point likert scale, scaling from extremely poor to extremely good. The study also recorded responses from the participants on innovative ways to manage finance in SMEs from the perspective of sustainability and big data, and even their attitude toward the concept of innovative finance on the 5-point likert scale. Lastly, the study also recorded the responses of the respondents on adapting to innovative technologies and big data within SMEs that will enhance the sustainability on a 5-point likert scale.

## Results and Discussion

The current study confirmed the following findings. Table 3 records the demographic details of the respondents, who participated in the structured questionnaires. When analyzing the data of SMEs, it was understood that 65.75 of them are males and 35.1 of them are females, spread across business units like manufacturing and services, fashion houses, units of handicraft and boutique. 52.01% of the respondents come under the manufacturing businesses and 47.12% of them belong to service units like beauty parlor, fast food, book shops and so on. Likewise, 40.10% of the SMEs were established for almost more than 10 years and come in between 15 years, 22.09% of the SMEs were established for more than 15 to 20 years, 19.61% of the SMEs were established for more than 20 years, and least 18.12% of them were established for <10 years. With regard to employee count, 49.21% of them disclosed high level of engagement in operations, 39.41% them disclosed a mediating level of employee engagement, and 13.01% exhibited low levels of employee engagement. Lastly, with regard to the employee revenue, 42.60% of them earned almost 5–10 million in a year, followed by 32.23% (<5 million) and 27.21% (<10 million).

**Table 3:** Demographic details of the respondents.

**Details**		**Number**	**Percentage %**	**Total count**
Gender	Male	920 (M)	65.7%	
	Female	480 (F)	35.1%	
Business nature	Manufacturing	820	52.01%	
	Services	580	47.12%	
Establishment year	<10	242	18.12%	1,400
	10–15 years	561	40.10%	
	15–20 years	322	22.09%	
	>20 years	272	19.61%	
Employee count	>150	172	13.01%	1,400
	25–150	687	49.21%	
	<25	536	39.41%	
Revenue	>10 million	371	27.21%	1,400
	5–10 million	582	42.60%	
	<5 million	442	32.23%	

**Figure 2:**
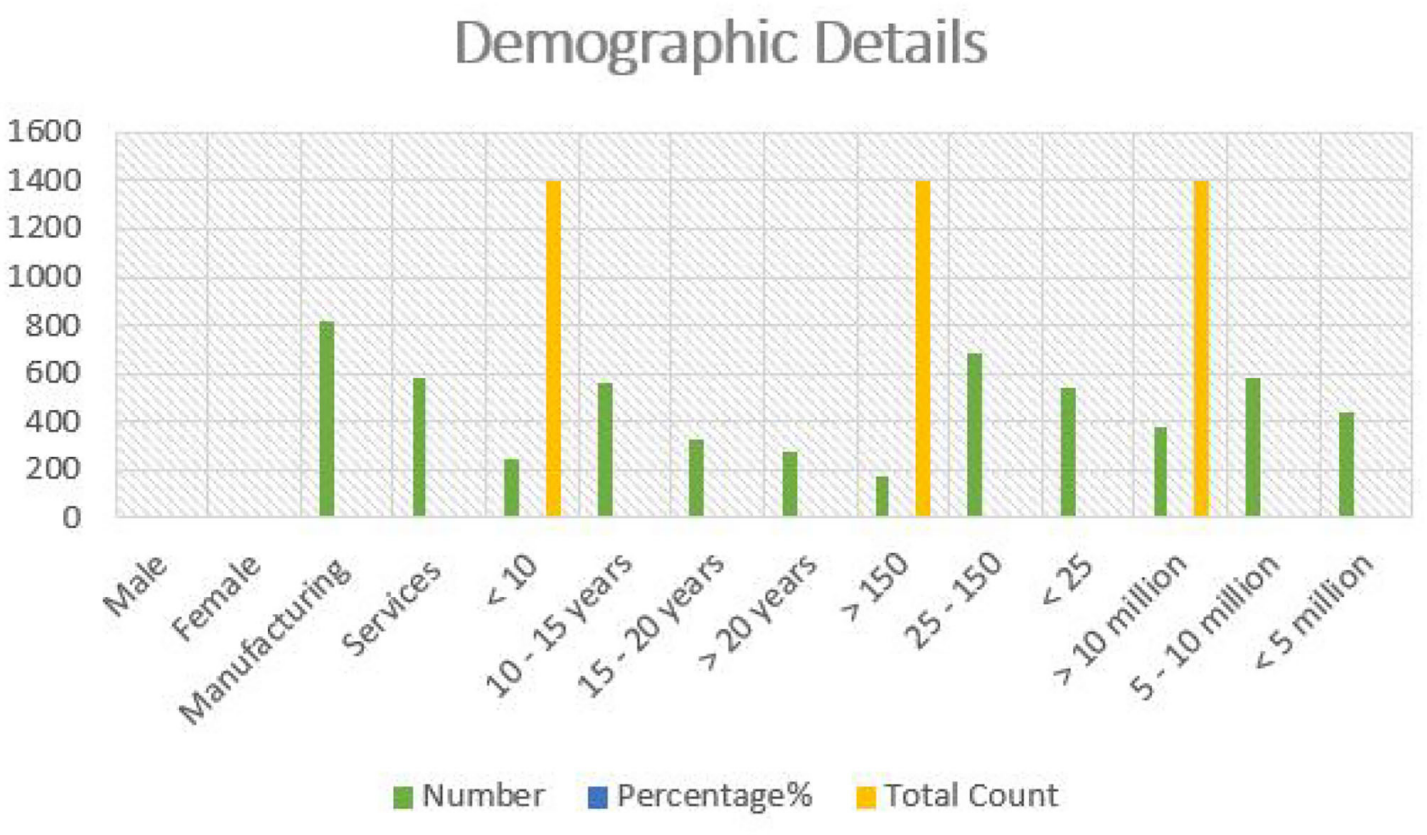
Graphical representation of demographic details of respondents.

The validation of the instrument used in the study is recorded in Table 4. From the table, it can be understood that the skewness test range results suggest that innovative finance, adapting technology within SMEs from the perspective of big data and sustainability are equally distributed. The extreme loading values presented in the table state that the instrument used is highly reliable and valid, which is calculated as 0.7. in terms of implementation. Moreover, the results presented in Table 4 also confirm the absence of multicollinearity in the data gathered. Figure 2 presents the graphical representation of validating the Instrument used in the study.

**Table 4:** Validating the instrument used in the study.

	**FI**	**AT**	**SME**
**FI** 1	0.921		
**FI** 2	0.882		
**FI** 3	0.945		
**FI** 4	0.891		
**FI** 5	0.820		
**AT** 1		0.991	
**AT** 2		0.891	
**AT** 3		0.912	
**AT** 4		0.890	
**SME** 1			0.893
**SME** 2			0.882
**SME** 3			0.960
**SME** 4			0.921
**SME** 5			0.881

**Figure 3:**
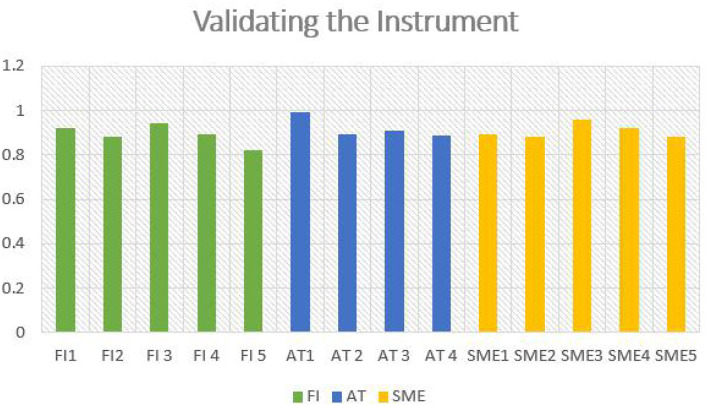
Graphical representation of validating the instrument used in the study.

The result of the empirical study shows that innovative ways to manage finance within the SMEs will play a crucial role from the perspective of SME sustainability, as the coefficient value seems to be higher than the cut-off value. Likewise, the results associated with variable adopting technology within the SMEs also confirmed that they seem to significantly impact the smooth functioning of the SMES, especially when advanced technologies are implemented in line with SME sustainability and big data. Al the identified variables seem to efficient and hence the results can be considered as reliable. The findings of the study also disclosed that there is no reliability and validity issue with regard to the identified constructs. The Figure 3 shows the Graphical Representation of Validating the Instrument used in the study.

**Figure 4:**
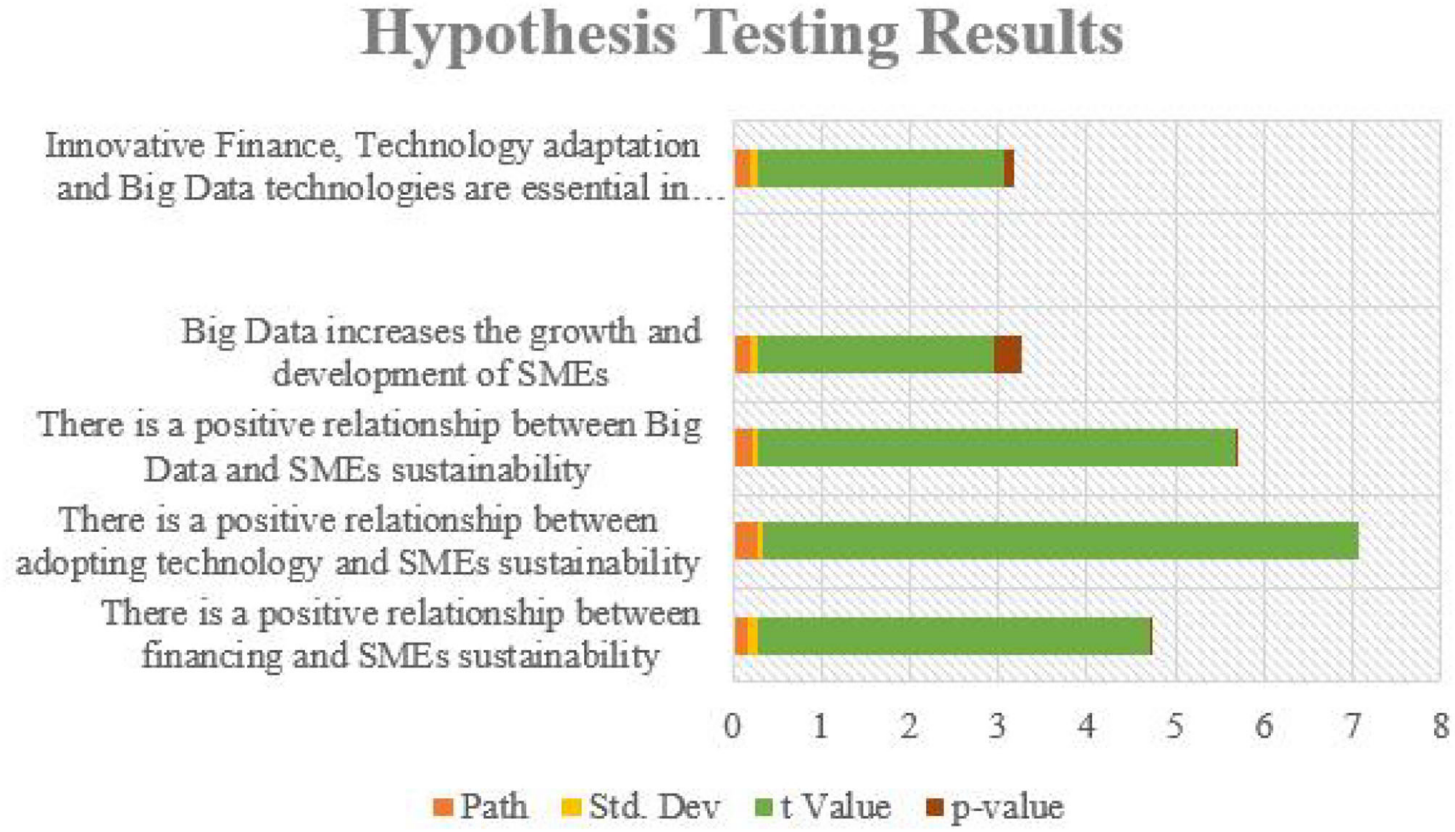
Graphical representation of results of hypothesis testing.

In addition to, the study presented the results in measurement models including all the identified various variables. Table 5 presented the measurement model results of the study, which indicate that there exists a relationship between the constructs–innovative finance, adopting technology within the SMES, adapting to big data technologies from the perspective of SME sustainability and big data. Figure 4 represents the results of Hypothesis testing.

**Table 5:** Results of hypothesis testing.

**Relationships**	**Path**	**Std. Dev**	***t* Value**	***p*-value**
**There is a positive relationship between financing and SMEs sustainability**	0.169	0.121	4.425	0.002
**There is a positive relationship between adopting technology and SMEs sustainability**	0.275	0.071	6.718	0
**There is a positive relationship between Big Data and SMEs sustainability**	0.212	0.081	5.387	0.014
**Big Data increases the growth and development of SMEs**	0.198	0.079	2.671	0.31
**Innovative Finance, Technology adaptation and Big Data technologies are essential in SMEs sustainability**	0.19	0.08	2.781	0.124

The study found that the hypothesis proposed in the study have high impact on the SME sustainability. Results of the study document that there is a positive relationship between SME and innovative finance, adapting to technological innovations, adapting to big data technologies, especially from the perspective of SME sustainability and big data. The study also found that the SME in China are high in number and hence the operations of the SMEs should focus on adopting to innovative finance, technology from the perspective of big data and sustainability, which stay significant for the survival of SMEs in this modern world. Even big data technologies will create new challenges and opportunities to the SMEs in contemporary world. Furthermore, there is a positive relationship between adopting technology and SMEs sustainability, which will expose SMEs to new opportunities and challenges. In short, it can be summarized that Innovative Finance, Technology adaptation and Big Data technologies are essential in SMEs sustainability.

## Conclusion

The present article has proposed and tested the relationship between Innovative Finance, Technology adaptation and Big Data technologies, which play an essential role in SMEs sustainability. Hence this study examined the financial budgets of technology-based SMEs from the perspective of sustainability and big data. The results of the study indicated that there is a positive relationship between SME and innovative finance, adapting to technological innovations, adapting to big data technologies, especially from the perspective of SME sustainability and big data. There is a positive relationship between adopting technology and SMEs sustainability, which will expose SMEs to new opportunities and challenges. Thus, it can be argued that can be summarized that Innovative Finance, Technology adaptation and Big Data technologies are essential in SMEs sustainability.

## Data Availability Statement

The original contributions presented in the study are included in the article/supplementary material, further inquiries can be directed to the corresponding author/s.

## Author Contributions

All authors listed have made a substantial, direct, and intellectual contribution to the work and approved it for publication.

## Conflict of Interest

The authors declare that the research was conducted in the absence of any commercial or financial relationships that could be construed as a potential conflict of interest.

## Publisher's Note

All claims expressed in this article are solely those of the authors and do not necessarily represent those of their affiliated organizations, or those of the publisher, the editors and the reviewers. Any product that may be evaluated in this article, or claim that may be made by its manufacturer, is not guaranteed or endorsed by the publisher.
